# Incidence and Outcomes of Postoperative Atrial Fibrillation after Coronary Artery Bypass Grafting of a Randomized Controlled Trial: A Blinded End-of-cycle Analysis

**DOI:** 10.31083/j.rcm2304122

**Published:** 2022-04-01

**Authors:** Ahmad Farouk Musa, Jeswant Dillon, Mohamed Ezani Md Taib, Alwi Mohamed Yunus, Abdul Rais Sanusi, Mohd Nazeri Nordin, Julian A. Smith

**Affiliations:** ^1^Jeffrey Cheah School of Medicine & Health Sciences, Monash University Malaysia, 47500 Subang Jaya, Selangor, Malaysia; ^2^Victorian Heart Institute, Monash University, 3168 Melbourne, Australia; ^3^Department of Cardiothoracic Surgery, National Heart Institute, 50400 Kuala Lumpur, Malaysia; ^4^Department of Surgery, School of Clinical Sciences at Monash Health, Monash University, 3168 Melbourne, Australia; ^5^Department of Cardiothoracic Surgery, Monash Health, 3168 Melbourne, Australia

**Keywords:** Tocovid, postoperative atrial fibrillation (POAF), CABG, CICU stay, total hospital stay, morbidity, mortality

## Abstract

**Objective:**

The objective of this study is to analyse the incidence of postoperative atrial fibrillation (POAF), demography, post-operative outcomes including morbidity and mortality, length of Cardiac Intensive Care Unit (CICU) stay, High Dependency Unit (HDU) stay, and total hospital stay in patients undergoing coronary bypass grafting (CABG) at Institut Jantung Negana (IJN).

**Methods:**

We conducted a prospective, randomised, controlled trial. We supplied the treatment group with Tocovid capsules and the control group with placebo containing palm superolein.

**Results:**

Since January 2019, we have recruited the target population of 250 patients. However, the result is still blinded as we are still analysing blood samples for tocotrienol levels. 89.2% of patients completed the study with a 3.6% mortality and a 7.6% attrition rate. 35.2% of the patients developed POAF, the mean time being 46.06 ± 26.96 hours post-CABG. We did not observe any statistically significant difference when we compared left atrial size, New York Heart Association (NYHA) functional class, ejection fraction and premorbid history, besides EuroSCORE II (The European System for Cardiac Operative Risk Evaluation II) status except for older age group, right atrial size, and pleural effusion. There was also no difference in bypass time, cross clamp time or number of anastomoses. However, we noted a significant difference in death (*p* = 0.01) and renal failure requiring dialysis (*p* = 0.007) among patients with POAF; those patients also had a longer CICU stay (*p* = 0.005), HDU stay (*p* = 0.02), and total hospital stay (*p* = 0.001).

**Conclusions:**

POAF is associated with a higher incidence of renal failure and death while it increases CICU, HDU, and total hospital stay. It remains to be seen whether Tocovid reduces POAF and its associated sequelae.

**Clinical Trial Registration:**

NCT03807037 (Registered on 16 January 2019).

## 1. Introduction

One of the commonest complications of cardiac surgery is postoperative atrial fibrillation (POAF). It occurs in about 20% to 40% [1] of patients after isolated coronary bypass grafting (CABG) and more often after combined CABG and valve surgery [2]. In an earlier retrospective study [3], we observed a prolonged Cardiac Intensive Care Unit (CICU) stay, High Dependency Unit (HDU) stay and total hospital stay among these patients, with a projected increase in resource utilisation. This was accompanied by a statistically significant increase in the incidence of stroke and death [3].

There is no single unifying mechanism in the development of POAF. It is generally agreed that both a susceptible substrate and a trigger factor are needed to initiate POAF [4, 5]. While multiple factors may initiate POAF, current data suggests that the postoperative inflammatory state after CABG plays a significant role in initiating POAF [6, 7]. More precisely, it is the shed mediastinal blood that serves as a notable source for this inflammation [8, 9, 10]. Recent data also showed that the incidence of POAF increased when the pericardium was opened. In contrast when the pericardium remained intact, as in transcatheter aortic valve replacement, a risk reduction of 82% was observed [11, 12].

It is currently believed that besides the inflammatory milieu, the presence of oxidative stress also predisposes to POAF [13]. This happens when the reactive oxygen species produced inundates the endogenous antioxidant defences [14]. When the recruited leukocytes are activated to release $O_{2}$ (superoxide) by reducing oxygen at the expense of nicotinamide adenine dinucleotide phosphate (NADPH), oxidative stress ensues [15]. The occurrence of a highly inflammatory and pro-oxidant state which generally takes place after CABG predisposes to the development of POAF [16]. As described above, the intrapericardial inflammation and oxidative stress trigger POAF through some pathological pathways originating from shed mediastinal blood following CABG [17].

In light of this pathogenesis, we postulate that using a potent anti-inflammatory antioxidant might mitigate POAF. We decided on tocotrienol, a compound that has not been commonly investigated, despite its much superior antioxidative and anti-inflammatory effect compared to its cousin, tocopherol [18]. We hypothesise that this may confer a therapeutic advantage in the safety endpoints post-CABG by reducing the incidence of POAF and its adverse sequelae.

## 2. Aims

To determine the incidence of POAF, demography, post-operative outcomes including morbidity and mortality, length of CICU stay, HDU stay, and total hospital stay.

## 3. Methods

### 3.1 Study Design

We designed this study as a prospective, double-blind, randomised, controlled trial involving parallel groups. All patients who were admitted at the Institut Jantung Negana (IJN), Kuala Lumpur, for CABG, or CABG and valve surgery, were automatically recruited into the study.

Patients were divided into two arms using a computer-generated randomisation programme: (1) a Control group with placebo plus standard care, and (2) a Treatment group with Tocovid, plus standard care.

At least two days prior to surgery, the blinded randomised patients were administered daily with either 400 mg Tocovid in two divided doses, or placebo, which was prepared by Hovid Berhad, a company based in Ipoh, Malaysia. Each 200 mg soft-gel Tocovid capsule contained a cocktail of tocotrienol (61.52 mg alpha-Tocotrienol, 112.80 mg gamma-Tocotrienol and 25.68 mg delta-Tocotrienol) and tocopherol (91.60 IU alpha-tocopherol). This regime was continued post-CABG until six weeks follow-up when the study was terminated.

We decided on giving 400 mg of Tocovid daily since many other clinical studies [19, 20] have used this regime without any adverse effects. We continued the treatment until the patient was discharged. The patients and the surgeons were blinded throughout the study, as were the research assistants. Only Clinical Research Nurses were not blinded. Onsite cardiothoracic ward nurses monitored and ensured compliance. Postoperatively all patients were observed for any electrocardiogram (ECG) changes via continuous ECG monitoring. All POAF episodes were treated according to the preference of the attending cardiothoracic surgeon.

**Fig. 1.**
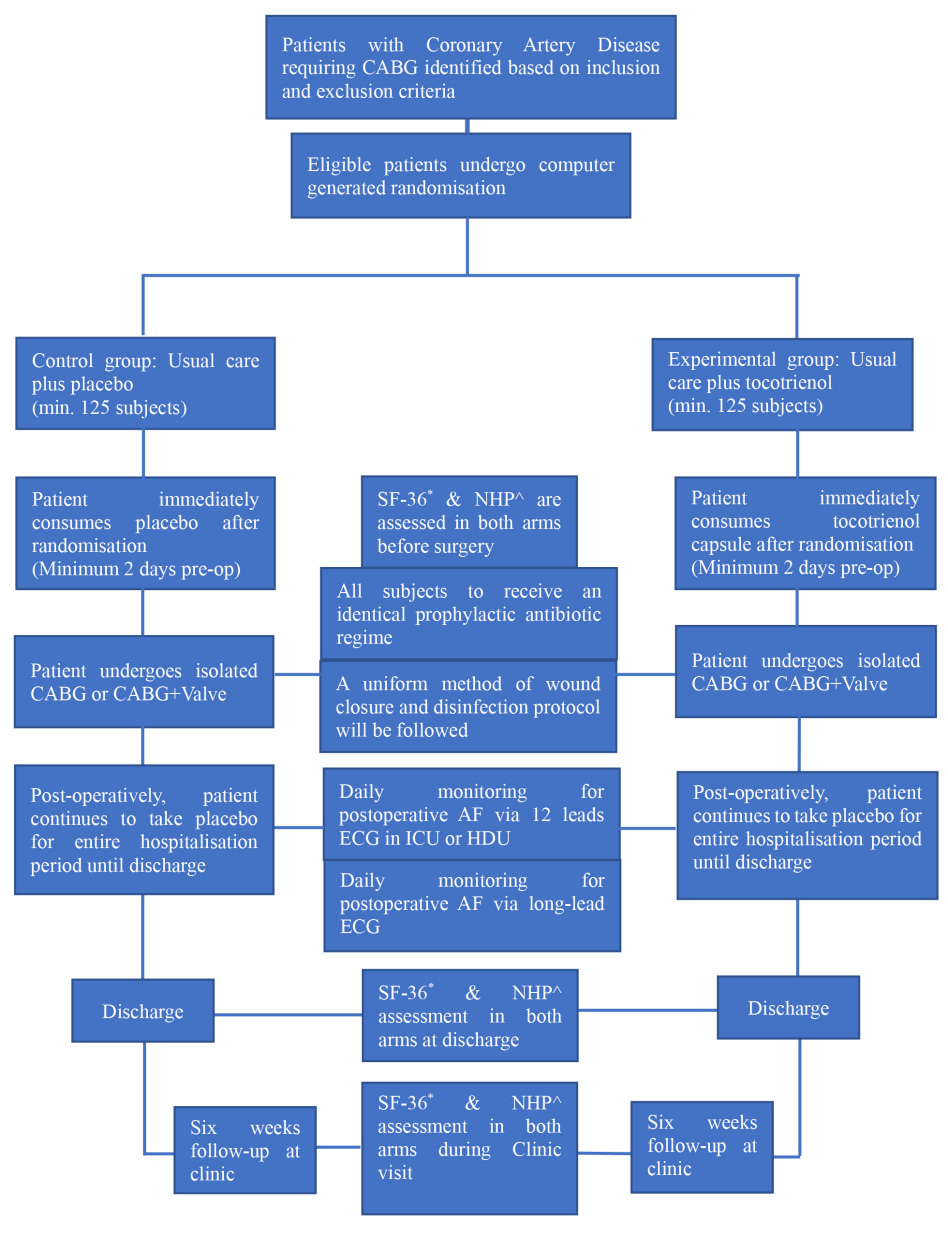
**Study Flow Chart**. *SF-36, Short Form 36 Questionnaire; ^NHP, Nottingham Health Profile Questionnaire.

For the study flow chart, refer to attachment: Fig. 1.

### 3.2 Inclusion and Exclusion Criteria

Inclusion Criteria:

Males or females over 18 years of age.

Elective, on-pump surgery of coronary artery revascularisation, either isolated or combined with valve surgery.

Exclusion criteria:

Urgent or emergency surgery as well as off-pump surgery.

Poor left ventricular (LV) function (ejection fraction (EF) <30%).

Allergy to palm oil or Vitamin E, or any form of arrhythmia pre-operatively.

Long-term treatment with corticosteroid.

Participation in other clinical trial within three months prior to the study.

Vitamin E or other potent antioxidant supplementation with within one month prior to randomisation.

### 3.3 Study End Points

The primary end point was POAF occurrence as confirmed on an ECG by the absence of p-wave and irregularly QRS complex of at least 30-second duration. For shorter ECGs, we diagnosed atrial fibrillation (AF)/atrial flutter (AFL) on the arrhythmia present at onset or termination [21]. The secondary end points were the length of hospital stay (LoHS) including both CICU and HDU stay.

### 3.4 Sample Size Calculation

For sample size calculation, we used the PS Power and Sample Size Calculation Software (Version 3.1.6, Developer: W.D. Dupont & W.D. Plummer. Licensed under a Creative Commons Attribution–NonCommercial-NoDerivs 3.0 United States License).

We estimated sample size based on findings from a prior study by Musa *et al*. [3]. The researchers found POAF incidence at IJN to be 28.7%. Assuming the true relative risk of AF for experimental subjects relative to controls is 0.45 [22], then if we use the PS Power and Sample Size Calculator [23, 24] with α equivalent to 0.05 and power (1-β) of 0.8, the estimated sample size is 103 in each arm. Assuming a possible attrition rate of 20%, our total sample size is: 103 + 20 (103) × 2 = 250 subjects, with 125 controls and 125 experimental subjects.

### 3.5 Statistical Analysis

For statistical analysis of the data, we used the SPSS software version 27.0 (IBM Inc., Chicago, IL, USA).

## 4. Ethics Declaration

We conducted this study according to the Malaysian Good Clinical Practice Guideline while abiding by the Helsinki Declaration revised in 2013. Informed written consent was obtained from all subjects prior to their participation in this study.

We sought ethics approvals from three institutions: the Institut Jantung Negara Research Ethics Committee (IJNREC/201/2017), the Monash University Human Research Ethics Committee (MUHREC) (2017-9227-10263) and the National Pharmaceutical Regulatory Agency (NPRA) (CTX-180304). The study was registered with the National Medical Research Register (NMRR-17-1994-34963) and the US National Library of Medicine-Clinical Trials (NCT03807037).

## 5. Results 

Recruitment of patients started on 21 January, 2019 and we reached 250 patients on 30 June, 2021 from a total of 1128 patients screened within that period. The current results are based on patients’ data; these were retrieved from patients who have completed the study. The tabulated figures consist of data extracted from both the IJN computer system and physical records.

Our population sample consists of 223 patients (89.2%) who have completed the study. There were 11 withdrawals and another 7 were lost during follow-up. We recorded 9 deaths (3.6%) which was slightly lower compared to our earlier study [25] in 2018 where the mortality rate stood at 4.66%. Therefore, the attrition rate was 7.2%. Eighteen serious adverse events (SAE) were reported but none was related to the investigational product.

The statistical analyses on the results of our study below were on the non-unblinded dataset since we have not completed the collection of all the data. Hence, our analyses were between the POAF group against the non-POAF group–not Tocovid versus control. This final analysis will only be conducted at the end of the study; at that time, all the data would have been completed and the groups unblinded. We also wish to emphasize that our analyses were restricted to patients with complete data. Hence, there might be some variations with regard to the number of patients analysed in each section.

### 5.1 Patients’ characteristics

Table 1 below describes the characteristics of our study sample. The mean age was 60.88 ± 7.79 ranging between 39 to 85 years old. Older age group is associated with POAF and this consolidated the fact that age has always been considered the most consistent factor responsible for an increased incidence of POAF [26]. In fact, it is an independent predictor of POAF [27], the higher occurrence of POAF in elderly patients due to age-related comorbidities [28] where ageing causes degenerative changes in the atrium as well as changes in the atrial physiology. These changes were described by Amar *et al*. [29] as having shorter refractory period, delayed sinoatrial (SA) and atrioventricular (AV) nodal conductivity, atrial stiffening and spluttering of the arial waveform. Cardiac surgery could also cause injury to the sympatho-vagal fibres of the cardiac plexus of elderly patients, leading to POAF [30]. Indeed, Mathew *et al*. [31] noted a 75% increase in the odds of developing POAF in every ten-year increase in age.

**Table 1.** **Characteristics of our sample population and their association with POAF**.

Demographic		Total, mean ± SD/n (%)	Non-POAF group, mean ± SD/n (%)	POAF group, mean ± SD/n (%)	*p*-value
Age (years)		60.88 ± 7.79	59.93 ± 8.54	62.36 ± 6.20	0.02*
Range: 39 to 85 years old					
Gender:					${\mathrm{0.20}}^{a}$
	Male	196	121 (61.7)	75 (38.3)	
	Female	46	33 (71.7)	13 (28.3)	
Population:					${\mathrm{0.40}}^{a}$
	Malay	199	122 (61.3)	77 (38.7)	
	Chinese	8	6 (75.0)	2 (25.0)	
	Indian	34	25 (73.5)	9 (26.5)	
	Other	1	1 (100.0)	0 (0.0)	
EuroSCORE II:					${\mathrm{0.39}}^{a}$
	Low risk	102 (42.7)	66 (64.7)	36 (40.9)	
	Medium risk	108 (45.2)	70 (64.8)	38 (35.2)	
	High risk	29 (12.1)	15 (51.7)	14 (48.3)	

*p-value significant at 0.05 using independent *T*-test. ^a^Test using Chi-Square test.

As a predictive factor of POAF, gender remains a contentious matter. While it is controversial to assume that male gender is a predictor of POAF, the effect of gender on POAF is definitely an interesting field of study. It has been shown that the female gender is shielded against POAF [32] but other studies have shown poorer outcomes among women after CABG [33, 34, 35, 36]. Meanwhile, Filardo *et al*. [37] attested to the poorer late survival in both women and men; however, they also showed that the early burden of POAF was less in women. While the debate continues, our results showed that there was no statistically significant difference between the sexes in developing POAF although the majority (80.9%) of our patients were males.

However, we found no statistically significant difference exists between the different ethnic groups in terms of POAF. However, this is a marked difference from our previous study [25] in which the Indian population had significantly lower odds of developing POAF compared to the other races. It also contrasts with a Singaporean study [38] that found that Malays and Chinese, as compared to Indians, had a higher likelihood of developing this phenomenon post-CABG. Western papers [39, 40, 41] showed that Caucasians were more prone to developing POAF compared to blacks, and genetic disparity between the races was thought to be the reason [39]. However, the survival rate of POAF patients after CABG showed that the black race was a significant predictor for decreased survival [40, 41]. This finding was definitely useful to both the surgeons and their patients.

Our study cohort showed that most of our patients (87.9%) were in the low and medium risk EuroSCORE II group. We observed no statistically significant difference in the development of POAF in between these groups. However, an earlier study by Chen-Scarabelli *et al*. [42] showed that higher EuroSCORE was associated with POAF though not with mortality after CABG surgery.

### 5.2 POAF Characteristics of Study Patients

Table 2 below refers to the POAF (post-operative AF) characteristics of our study patients. 35.2% of patients developed POAF which is higher compared to our previous study [25] where 28.7% of the patients developed AF post CABG. However, it would be hasty to conclude that Tocovid has no effect in lowering the POAF rate since this is still a blinded analysis. Nevertheless, 35.2% falls within the POAF incidence range cited in the literature [43, 44, 45].

**Table 2.** **Characteristics of POAF**.

Characteristics of AF		n (%), mean ± SD
Occurrence of POAF		88 (35.2)
Time from surgery to POAF (minutes)		2793.61 ± 1617.36
Range: 10 to 7044 minutes		
Duration (hours):		
	≤48	45 (52.9)
	>48	40 (47.1)
Number of episodes:		
	Single	45 (51.1)
	Multiple	43 (48.9)
Atrial fibrillation on discharge		0 (0.0)

The mean time for POAF development of was 46.56 ± 26.96 hours after surgery; in the second postoperative day, and was within the range cited by the literature of 2–3 days after surgery [46]. Its occurrence ranges from 20 minutes to 5.7 days post-CABG. However, this was also within the cited range in the literature [46, 47, 48] POAF mainly occurs within the first week post-surgery, with 70% of cases [49] within the first four postoperative days. In addition, we noticed a single episode of POAF in 51.1% of our patients; while the remaining 48.9% had multiple AF episodes post-CABG. Nonetheless, all study patients reverted without exception, and they were all discharged in sinus rhythm.

### 5.3 Pre-operative Characteristics of Study Patients

Malaysia has the highest rate of obesity and overweight among Asian countries according to World Health Organization (WHO) [50]. Table 3 below indicates that 52.22% of the study sample was overweight and 25.12% was obese according to the Asian guidelines [51]. However, no statistically significant difference was observed between the groups in terms of POAF occurrence. This finding contradicts some reports in the literature [52, 53, 54, 55] which suggest that, compared to their non-obese counterparts, obese patients are more prone to develop POAF. An earlier work of Sun X *et al*. [56] together with a most recent study by Vural Ü and Aglar A [57] have suggested that obesity is a predictor for POAF. However, a very recent meta-analysis [58] of 36 prospective studies which has yet to be peer-reviewed found that obesity might not increase the risk of developing POAF after CABG.

**Table 3.** **Pre-operative characteristics and their association with POAF**.

Pre-operative characteristic		Total, mean ± SD/n (%)	Non-POAF group, mean ± SD/n (%)	POAF group, mean ± SD/n (%)	*p*-value
Body Mass Index (kg/$m^{2}$)		27.15 ± 4.39	27.25 ± 4.52	26.86 ± 4.39	${\mathrm{0.29}}^{a}$
	<18.5	2 (0.8)	0 (0.0)	2 (100.0)	
	18.5–22.9	57 (23.6)	38 (66.7)	19 (33.3)	
	23–29.9	124 (51.2)	79 (63.7)	45 (36.3)	
	≥30	59 (24.4)	37 (62.7)	22 (37.3)	
Range: 17.6 to 42.47					
New York Heart Functional Class:					${\mathrm{0.89}}^{a}$
	NYHA I	145 (60.2)	92 (63.4)	53 (36.6)	
	NYHA II	94 (39.0)	61 (64.9)	33 (35.1)	
	NYHA III	2 (0.8)	1 (50.0)	1 (50.0)	
	NYHA IV	0 (0.0)	0 (0.0)	0 (0.0)	
Left ventricular ejection fraction		51.45 ± 9.02	51.92 ± 8.94	50.67 ± 9.09	${\mathrm{0.29}}^{b}$
Range: 9 to 67					
Left atrial size (mm)		18.09 ± 4.96	17.63 ± 4.34	18.74 ± 5.85	${\mathrm{0.09}}^{b}$
Range: 9 to 46					
Right atrial size (mm)		13.88 ± 3.09	13.43 ± 2.62	14.51 ± 3.55	0.007*
Range: 7.7 to 31					

*p-value significant at 0.05 using independent *T*-test. ^a^Test using Chi Square test. ^b^Test using independent *T*-test.

We have excluded poor EF (<30%) from our study, so most patients were in New York Heart Functional Class (NYHA) I and II. According to our findings, although the mean left ventricular ejection fraction (EF) for the non-POAF group was slightly better at 51.92% as compared to the POAF group at 50.67%, this was not statistically significant. Additionally, although left atrial size was larger in the POAF group as compared to the non-POAF group, this was also not statistically significant. These two findings contradicted some of the literature [59, 60, 61, 62] that established a correlation between poor EF and an increased left atrial size in POAF development. But strikingly, the POAF group had a statistically significant dilated right atrial size as compared to the non-POAF group: this is consistent with the literature [63] where POAF is believed to be due to dilatation process and the ensuing remodelling process itself [63].

### 5.4 Medical History

The pre-morbid history of our patients is illustrated in Table 4 below. As expected, the majority were afflicted with hypertension (81%), diabetes mellitus (62%) and hypercholesterolaemia (90.1%) but we noticed no statistically significant difference between the groups. Similarly, our analysis on chronic kidney disease which is normally linked to POAF [64, 65], showed no significant difference in between the two groups.

**Table 4.** **Underlying medical conditions and their association with POAF**.

Medical condition		Total, n (%)	Non-POAF group, n (%)	POAF group, n (%)	$\chi^{2}$	*p*-value
COPD:					-	${\mathrm{0.7}}^{b}$
	Yes	3 (1.2)	2 (66.7)	1 (33.3)		
	No	239 (98.8)	152 (63.6)	87 (36.4)		
Asthma:					-	${\mathrm{0.64}}^{b}$
	Yes	1 (0.4)	1 (100.0)	0 (0.0)		
	No	241 (99.6)	153 (63.5)	88 (36.5)		
Hypertension:					0.35	${\mathrm{0.56}}^{a}$
	Yes	196 (81.0)	123 (62.8)	73 (37.2)		
	No	46 (19.0)	31 (67.4)	15 (32.6)		
Diabetes mellitus:					0.46	${\mathrm{0.49}}^{a}$
	Yes	150 (62.0)	93 (62.0)	57 (38.0)		
	No	92 (38.0)	61 (66.3)	31 (33.7)		
Hypercholesterolemia:					0.32	${\mathrm{0.57}}^{a}$
	Yes	218 (90.1)	140 (64.2)	78 (35.8)		
	No	24 (9.9)	14 (58.3)	10 (41.7)		
Chronic kidney disease:					0.08	${\mathrm{0.77}}^{a}$
	Yes	23 (9.5)	14 (60.9)	9 (39.1)		
	No	219 (90.5)	140 (63.9)	79 (36.1)		
Current or ex-smoker:					0.08	${\mathrm{0.77}}^{a}$
	Yes	127 (54.3)	83 (65.4)	44 (34.6)		
	No	107 (45.7)	68 (63.6)	39 (36.4)		
Alcohol intake:					-	${\mathrm{0.18}}^{b}$
	Yes	9 (3.9)	4 (44.4)	5 (55.6)		
	No	219 (96.1)	142 (64.8)	77 (35.2)		

^a^Test using Chi-Square test. ^b^Test using Fisher Exact Test.

When we compared current or ex-smokers with non-smokers to analyse the relationship between smoking habits and POAF incidence, we observed no statistically significant difference between them (*p* = 0.77). In contrast, a previous study [66] showed smokers tend to have a lower incidence of POAF (*p *< 0.05). However, the authors strongly recommended a ban on smoking for at least 4 weeks before surgery; in view of improving post-operative outcomes. The paper, however, might seem at odds with the presumption that nicotine increases the heart rate by stimulating the release of catecholamines and inducing the electrical instability of the atria by blocking the potassium currents, thereby increasing the risk of AF [67]. Another paper also found an association between smoking and AF-related outcomes such as bleeding, thrombosis and death [68]. Due to these conflicting reports, a meta-analysis which included 36 meta-analysis was conducted recently by Wan *et al*. [69]. They concluded that smoking was neither associated with an increased risk of POAF in CABG patients, nor does it have a protective effect.

### 5.5 Operative Details

We performed isolated CABG in 92.5% and CABG combined with valve surgery in 7.5% in our study population as shown in Table 5 below. Mitral valve replacement surgery accounts for almost 60% of cases of the combined surgery group; only one case involved mitral valve repair. Aortic valve replacement was performed in the remaining 40% of cases. However, no statistically significant difference was observed between these groups in terms of developing POAF. This contradicts the general belief that a combined valve with CABG would result in a higher occurrence of POAF [70, 71] besides the use of heart-lung bypass machine [72, 73]. In a recent multicentre prospective study [74] involving a total of 28 centres, it was reconfirmed that combined valve and CABG was significantly correlated with the occurrence of POAF.

**Table 5.** **Patient operative details (CABG, coronary bypass grafting) and their association with POAF**.

Operative details		Total, mean ± SD/n (%)	Non-POAF group, mean ± SD/n (%)	POAF group, mean ±SD/n (%)	χ^2^/*t*-value	*p*-value
Surgery type:						
	CABG alone	222 (92.5)	143 (64.4)	79 (35.6)	1.49	${\mathrm{0.22}}^{a}$
	CABG + valve	18 (7.5)	9 (50.0)	9 (50.0)		
Bypass time (in minutes)		97.00 ± 35.59	95.98 ± 38.43	98.91 ± 30.35		${\mathrm{0.54}}^{b}$
Range: 42 to 304 minutes						
Cross-clamp time (in mins)		75.84 ± 30.05	75.86 ± 32.28	75.81 ± 25.79	0.22	${\mathrm{0.99}}^{b}$
Range: 17 to 244 minutes						
Number of anastomoses:						
	Single	6 (2.5)	2 (33.3)	4 (66.7)	-	${\mathrm{0.13}}^{c}$
	Multiple	234 (97.5)	150 (64.1)	84 (35.9)		

^a^Test using Chi Square test. ^b^Test using Independent *T*-test. ^c^Test using Fisher exact test.

The mean cross-clamp time in our study was 75.84 ± 30 minutes and the mean bypass time was 97 ± 35.59 minutes. Similarly, no statistically significant difference was observed between the two groups though we knew that both the cross-clamp time and the bypass time had an association with POAF development [75, 76, 77, 78]. Similarly, we could not find any significant corelation between the number of distal anastomoses with POAF in our study with a similar finding observed in another study by Lotfi *et al*. [79].

### 5.6 Post-operative Outcomes

Adverse outcomes such as stroke, reoperation, infection, renal failure, respiratory complications and other cerebral insults, besides a twofold increase in mortality [80, 81, 82] were always associated with POAF. Admittedly, while this correlation might not be direct, it does contribute to the increase in morbidity and mortality post-CABG. In Table 6 below, although more POAF patients developed stroke as compared to non-POAF, it was not statistically significant. While the mortality rate in our study population was 3.6% as compared to the mortality rate in our earlier publication (4.66%) [25] this was definitely much lower. But we saw a statistically significant difference with a threefold increase in death among patients that developed POAF post-CABG. Our finding concurs with a recent paper by Emma Thorén *et al*. [83] where POAF was associated with mortality with a more recent paper from Taiwan which also concluded that in the Asian population, POAF is significantly correlated with an overall mortality [84].

**Table 6.** **Post-operative outcomes and their association with POAF**.

Post-operative outcomes		Total, n (%)	Non-POAF group, n (%)	POAF group, n (%)	χ ^2^	*p*-value
Stroke:					-	${\mathrm{0.14}}^{b}$
	Yes	4 (1.7)	1 (25.0)	3 (75.0)		
	No	232 (98.3)	148 (63.8)	84 (36.2)		
Sternal infection:					-	${\mathrm{0.6}}^{b}$
	Yes	6 (2.5)	4 (66.7)	2 (33.33)		
	No	232 (97.5)	146 (62.9)	86 (37.1)		
Respiratory problems:					-	${\mathrm{0.2}}^{b}$
	Yes	9 (3.8)	4 (44.4)	5 (55.6)		
	No	229 (96.2)	146 (63.8)	83 (36.2)		
Renal failure requiring dialysis:					-	0.007**
	Yes	12 (5.0)	3 (25.0)	9 (75.0)		
	No	226 (95.0)	147 (65.0)	79 (35.0)		
Endocrine problems:					-	${\mathrm{0.63}}^{b}$
	Yes	1 (0.4)	1 (100.0)	0 (0.0)		
	No	237 (99.6)	149 (62.9)	88 (37.1)		
Pleural effusion:					4.79	0.03*
	Yes	18 (7.6)	7 (38.9)	11 (61.1)		
	No	219 (92.4)	142 (64.8)	77 (35.2)		
Cardiac Tamponade:					0.16	${\mathrm{0.69}}^{a}$
	Yes	22 (9.2)	13 (59.1)	9 (40.9)		
	No	216 (90.8)	137 (63.4)	79 (36.6)		
Fever:					-	0.10^b^
	Yes	12 (5.0)	5 (41.7)	7 (58.3)		
	No	226 (95.0)	145 (64.2)	81 (35.8)		
Hyperkalaemia:					-	0.15^b^
	Yes	4 (1.7)	1 (25.0)	3 (75.0)		
	No	233 (98.3)	148 (63.5)	85 (36.5)		
Others:					-	0.06^b^
	Yes	7 (2.9)	2 (28.6)	5 (71.4)		
	No	231 (97.1)	148 (64.1)	83 (35.9)		
Death:					-	0.01*
	Yes	9 (3.75)	2 (22.2)	7 (77.8)		
	No	231 (96.25)	150 (64.9)	81 (35.1)		

*p-value significant at 0.05 using Chi Square Test. **p-value significant at 0.05 using Fisher exact Test. ^a^Test using Chi Square test. ^b^Test using Fisher Exact Test.

We also assessed other common complications as depicted in Table 6 but none of them, except pleural effusion, were significantly correlated with POAF. This is consistent with the finding by Brookes *et al*. [85] who found a link between new onset atrial fibrillation and pleural effusion. Similarly, Anderson [86] also showed that self-clearing chest tubes may reduce POAF although a randomized data is still needed to prove this claim. Interestingly a systematic review by Gozdek *et al*. [87] showed that posterior pericardial drainage to minimize any possibility of pericardial tamponade could reduce the odds of POAF by 58% indicating that POAF is possibly associated with pericardial collections. In our study cohort as well, we noticed that statistically significant number of patients with renal failure developed POAF. Similarly, the correlation between renal dysfunction and POAF has been established by Chua *et al*. [88]. While the mechanism was not fully understood, it was thought to be related to fluid overload and activation of renin-angiotensin-aldosterone cascade which would then lead to myocardial fibrosis [89].

### 5.7 Postoperative Stay

To date, no study in Malaysia has assessed the economic impact of managing POAF patients post cardiac surgery. The closest regional study was conducted in Thailand [90] where it was shown that there was a statistically significant high economic burden in managing POAF patients. Studies conducted elsewhere have demonstrated that POAF was associated with prolonged CICU stay and total hospital stay [91, 92]. This would of course be translated into an increased cost of hospitalisation. For instance, US patients who developed POAF would incur additional hospital treatment costs in the range of USD10,000 to USD20,000 [93]. Furthermore, US healthcare expenditures related to POAF management were approximately 1 billion USD per year [94]. Another study [95] demonstrated that POAF led to an increase in the utilisation of hospital resources, with an increase in the direct costs of managing affected patients. No such studies have been conducted locally regarding the financial burden in managing POAF patients. However, it would not be surprising if it yields similar results.

Based on the outcomes tabulated in Table 7 above, there was a statistically significant difference between the two groups with regard in to the mean duration of CICU stay (*p* = 0.005) and HDU stay (*p* = 0.02). Similarly, there was also a significant difference in total duration of hospital stay (*p* = 0.001). Our findings concur with one study [73] that collected data from 28 centres which showed that POAF occurrence was significantly correlated to the length of stay in CICU and total hospital stay with a resultant increase in resource utilisation.

**Table 7.** **The association between POAF and duration of CICU, HDU and total hospital stay, ventilation time and reintubation**.

Duration		Total, median ± IQR/n (%)	Non-POAF group, median ± IQR/n (%)	POAF group, median ± IQR/n (%)	*p*-value
Duration in CICU (minute)		1722 ± 2648	1632 ± 1638	2872 ± 5493	0.005*
Range: 640 to 67740 minutes					
Duration in HDU (minute)		1640 ± 1711	1545 ± 1510	2700 ± 2795	0.02*
Range: 190 to 14760 minutes					
Duration of ventilation (minute)		1134 ± 380	1110 ± 395	1190 ± 415	0.06^a^
Range: 350 to 17120 minutes					
Duration of hosp. stay (day)		7.0 ± 3	7.0 ± 2	8.0 ± 4	0.001*
Range: 5 to 86 days					
Reintubation:					0.12^b^
	Yes	8 (3.4)	3 (37.5)	5 (62.5)	
	No	230 (96.6)	148 (64.3)	82 (35.7)	

*p-value significant at <0.05 using Mann-Whitney Test. ^a^Test using Mann-Whitney test. ^b^Test using Fisher Exact test.

## 6. Discussion

35.2% of our study population exhibited POAF. This was slightly higher compared to our previous study [2] of about 28.7%. We should note that despite advances in the perioperative cardiac surgery care, the length of stay in the CICU, HDU, and the total hospital stay have remained unchanged over the years [96, 97]. At the moment we are unsure whether our prophylactic intervention using Tocovid would reduce the incidence of POAF in the study arm. Despite this uncertainty, we take comfort in the words of Sir William Ramsay, the Chemist Nobel Laureate, who reminds us that progress is made by trial and failure. Therefore, we shall endure until the study is unblinded.

As we outlined earlier, oxidative stress and inflammation from the shed mediastinal blood within the pericardium are now thought to be responsible for the pathogenesis of POAF. Cardiac surgery itself inflicts a trauma on the heart, and this is compounded by the use of cardiopulmonary bypass that produces ischaemic injury. Oxidative stress and the production of pro-inflammatory molecules from reperfusion injury after cardioplegic arrest activateds the production of leucocytes, nitrous oxide and reactive oxygen species [79, 98]. Human studies have demonstrated that a correlation exists between the development of POAF with systemic inflammation and oxidative stress [99, 100].

Studies have shown that longer CICU and hospital stay, and a higher rate of readmission were associated with POAF [95, 101]. These outcomes translate into approximately USD 2 billion out of more than USD 6 billion per year related to POAF care in the US [102, 103]. Unfortunately, no financial data from IJN of Kuala Lumpur or any other cardiac centres in Malaysia are available with regard to the total cost in managing patients with POAF. Nonetheless, there is no doubt that managing such patients entails higher costs. Therefore, reducing POAF incidence among post-CABG patients would benefit not only patients or hospitals but to the national economy itself.

We are aware that other compounds have been used in research to prevent POAF; for instance, polyunsaturated fatty acids (PUFAs), vitamin C, or a combination of vitamins C and E [104]. PUFAs have been demonstrated to reduce cardiovascular morbidity in animal models [105]. In a study by Rubanenko O and Rubanenko A [106], patients treated with PUFAs not only displayed reduced in inflammation and oxidative stress after CABG, but also exhibited a reduction in POAF after CABG. A meta-analysis [107] of 19 randomised controlled trials (RCTs) conducted in 2017 and a meta-analysis [108] in 2018 that included 14 RCTs also showed that CABG patients treated with PUFAs displayed a significant reduction of POAF compared to controls. In all these cases, an antioxidant prevented POAF, and the results were very promising the more so since our study is based on the same presumption.

Similarly, vitamin C, a known antioxidant, has been studied. A 2016 meta-analysis [109] of 7 RCTs showed that vitamin C treatment reduces the incidence of POAF. However, a more recent RCT [110] in 2018 found no significant difference. But to date, there are no guidelines on the use of vitamin C for POAF prophylaxis. A 2013 study [111] used combined antioxidants with vitamin C, vitamin E and PUFAs, demonstrated a significant reduction in the incidence of POAF among patients receiving these cocktails as compared to controls. However, to date, there are no guidelines regarding this protocol.

With the aforementioned studies and the postulated inflammatory and oxidative pathways in the promotion of POAF, we anticipate that using Tocovid, a strong antioxidant and anti-inflammatory agent, might be useful in mitigating the occurrence of POAF. We understand that it is still too early to make any definite claim. Nonetheless, this study has a scientific rationale, and once completed, it will be unblended for analysis.

## 7. Limitations

Our main limitation was the patients’ recruitment. This is due to the current COVID-19 pandemic which reduced the number of patients that could be enrolled in our study as a result of the limited availability of ICU beds. Similarly, the rate of tracing the patients’ medical records at the IJN Record Office dampened the pace of the study since only limited records were available for tracing each week. Not all the required data points were available on the track care, making reference to patients’ medical records unavoidable.

## 8. Conclusions

As a preliminary conclusion, we would like to reiterate that POAF after cardiac surgery was the most common complication after CABG; it occurred in 35.2% of our study population. There was a statistically significant difference among POAF patients with regard to the occurrence of renal failure and death; we observed a three-fold increase in both. Both CICU and HDU time, and also the total hospital stay, were significantly longer among POAF patients. This translates to a heavier economic burden on the patient, the hospital, and the economy, although we have not conducted any cost analysis in this study.

## Availability of Data and Materials

Harvard Dataverse: Replication Data for: A mid-cycle analysis on the role of Tocovid, a tocotrienol-rich vitamin E, in preventing atrial fibrillation after coronary artery bypass grafting (CABG) surgery.

Link: https://doi.org/10.7910/DVN/BZBHGX.

This project contains the following underlying data:

Set 1: Raw Data.

Set 2: Output Data.

CONSORT Checklist.
